# OA27 A Thrombus Tale

**DOI:** 10.1093/rap/rkae117.027

**Published:** 2024-11-05

**Authors:** Nandita Pai, Samundeeswari Deepak, Kishore Warrier, Satyapal Rangaraj

**Affiliations:** Queens Medical Centre, Nottingham, United Kingdom; Queens Medical Centre, Nottingham, United Kingdom; Queens Medical Centre, Nottingham, United Kingdom; Queens Medical Centre, Nottingham, United Kingdom

## Abstract

**Introduction:**

Non catheter related arterial thrombosis is very rare in children. Our patient had a peripheral arterial thrombus with no risk factors apart from a background of recurrent Henoch-Schönlein Purpura (HSP). He had no history of trauma. Thrombosis as a complication of HSP is extremely rare with onlyeight case reports so far in children and among those eight, there is only one resembling our case with an arterial thrombus. We considered other causes of thrombosis and screened our patient for thrombophilia, infection and malignancy.

**Case description:**

Our patient, a 12-year-old boy with a background of recurrent HSP for the last two years, presented with a rash on the left 2^nd^ toe followed by blackish discolouration of the distal part of the same toe, a few days later.

His initial presentation with purpuric rash, arthralgia, and abdominal pain was diagnosed as HSP and he required no intervention required for this. He continued to have regular flares of his HSP every six weeks, and these were self-limiting. His last flare was in February 2024.

In April 2024, his feet appeared dusky and mottled. He presented to his local hospital with a rash on the tip of his left 2^nd^ toe which turned gangrenous. He was otherwise systemically well. He was initially treated with antibiotics and acyclovir as a swab from the lesions grew staphylococcus aureus and the rash was bullous initially. He was referred to our tertiary rheumatology and haematology teams at Nottingham due to worsening ischemia and an absent left dorsalis pedis pulse.

His examination revealed normal blood pressure, mottled, dusky lower legs, warm left foot with a faint petechial rash near left malleolus, absent left Dorsalis pedis pulse and an ischemic tip of left 2^nd^ toe. Bilateral femoral and upper limb pulses were well felt. His respiratory, cardiovascular, abdomen & musculoskeletal examination were normal apart from an antalgic gait.

His clotting and thrombophilia screen including antiphospholipid screen was normal. His D dimer was raised at 1894ug/l. A duplex scan of his left leg showed occlusions in the distal anterior and posterior tibial artery as well as in the dorsalis pedis artery with no emboli. His inflammatory markers and autoantibodies were normal. Infection screen including mycoplasma, CMV, EBV and ASOT titres were all negative. Echo was normal. A skin biopsy from the toe revealed IgA vasculitis.

**Discussion:**

We had a multidisciplinary team (MDT) discussion with haematology, vascular surgery and dermatology. As per the MDT discussion, he was given a pulse of intravenous methylprednisolone after careful consideration weighing the risk versus benefit of steroids adding to the pro thrombotic state versus active vasculitis causing narrowing of the arteries. He was also commenced on Aspirin at 1 mg/kg/day. There was marked improvement in his symptoms with improvement in the discolouration and pain, his d-dimer reduced, and he was able to mobilise comfortably.

We felt that active vasculitis could be causing the vascular event. As immunology screen was negative and the skin biopsy was confirmatory of HSP. There was no thrombus specifically mentioned in the duplex but with D-dimer being raised it was a reasonable assumption was that there was a thrombus occluding the peripheral arteries.

As his HSP was recurring quite frequently and was bothersome, as well as the thought that the recurrences might contribute to further vascular events and complications, we started our patient on Azathioprine. We have also sent genetic tests and awaiting a catheter angiogram to complete the investigation process.

**Key learning points:**

• Consider vasculitis as one of the differentials in a child with unexplained venous or arterial thrombosis.

• In a child with recurrent HSP, keep an open mind for rare complications that may occur.

• Recurrent HSP in an older child may need immunosuppression.

• Treatment considerations in a case with vasculitis with a thrombus has to be in close connection with haematology to decide on anticoagulation and its implications on the young person and their lifestyle.

• Should there be any other investigations done or alternative diagnosis though of at this point?

